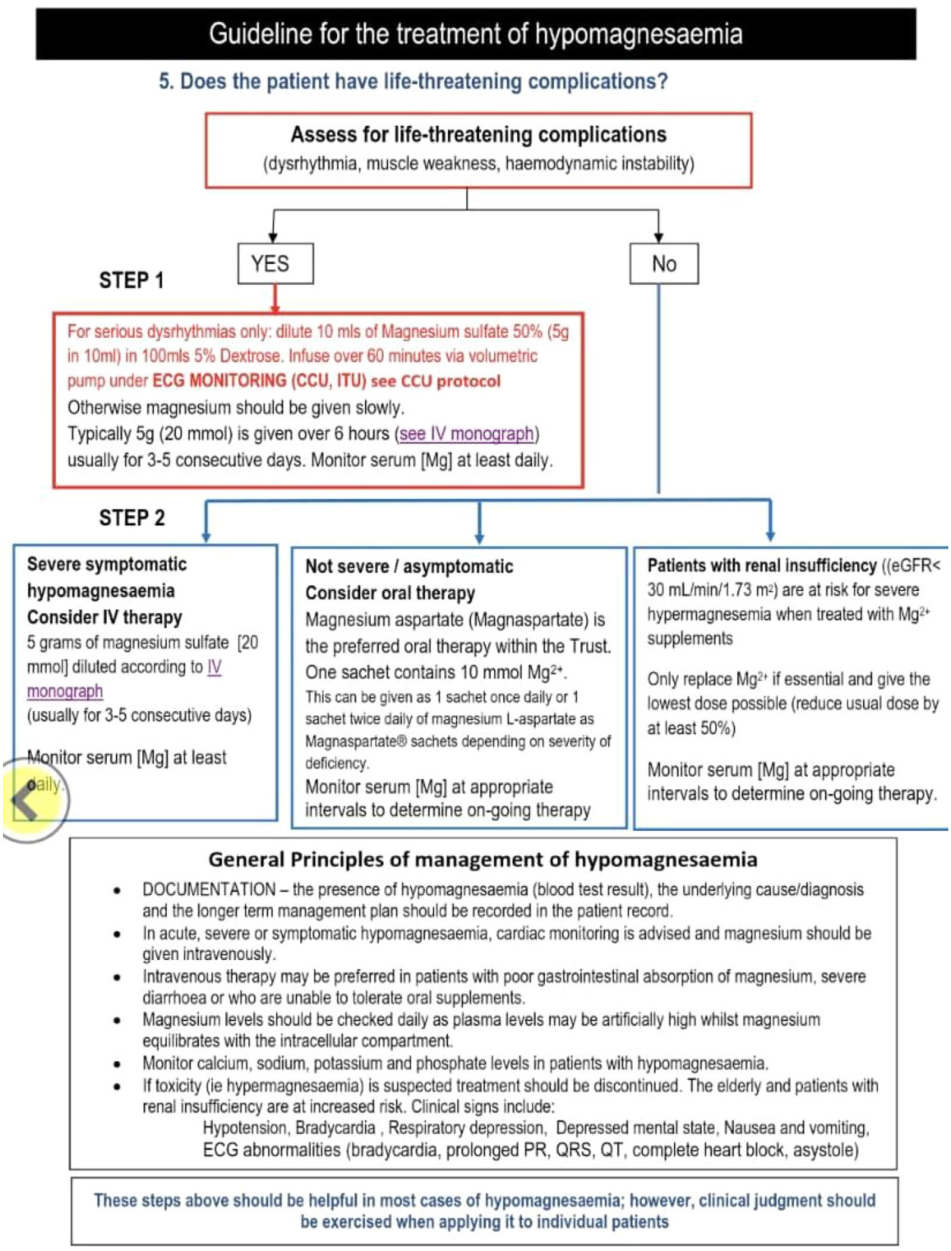# A clinical audit of hypomagnesaemia management at Scarborough General Hospital

**DOI:** 10.1016/j.fhj.2024.100207

**Published:** 2024-11-15

**Authors:** Dhanushan Gnanendran, Moses Grace Kintu, Aparna Ravikumar, Tadeusz Pawlak

**Affiliations:** Scarborough Hospital, Woodlands Dr, Scarborough, YO12 6QL, United Kingdom

## Abstract

**Background:**

Hypomagnesaemia is a common electrolyte disturbance that can lead to severe complications, including cardiac dysrhythmias and neuromuscular instability. Despite established guidelines by the York and Scarborough Teaching Hospitals NHS Foundation Trust, there is concern that these protocols are not consistently followed, potentially compromising patient outcomes. Objective: This audit aimed to evaluate the management of hypomagnesaemia at Scarborough General Hospital, focusing on adherence to trust guidelines and identifying areas for improvement in clinical practice.

**Methods:**

A retrospective analysis was conducted on 98 patients diagnosed with hypomagnesaemia between May and December 2023. Data on patient demographics, severity of hypomagnesaemia, ECG monitoring, treatment modalities and correction times were collected and analysed. The audit compared current practices against trust guidelines, specifically assessing the frequency of ECG monitoring, daily serum magnesium level checks, and the use of parenteral versus oral magnesium supplementation.

**Results:**

The audit revealed that 67% of patients had mild, asymptomatic hypomagnesaemia, while 33% presented with moderate to severe forms. ECGs were performed in 66% of cases, with 13.7% showing abnormalities. Despite guidelines recommending daily magnesium level monitoring, only 66% of patients received this care. Severe hypomagnesaemia was corrected within 3.5 days on average, while mild cases required 4.5 days. The frequent use of proton pump inhibitors was identified as a significant contributor to hypomagnesaemia. Notably, only half of the severe cases received the full recommended course of intravenous magnesium sulphate, with many being switched to oral supplementation prematurely.

**Conclusion:**

While some aspects of hypomagnesaemia management at Scarborough General Hospital align with trust guidelines, significant gaps remain, particularly in monitoring practices and adherence to treatment protocols. These findings highlight the need for targeted educational initiatives and potential revisions to existing guidelines to improve patient outcomes.

## Highlights

• About 67% of the audit patients presented with mild hypomagnesemia, and asymptomatic, pointing out the insidious but very prevalent nature of this condition.
• Although the guidelines recommended a three-day course of intravenous magnesium sulphate in severe cases, most received one dose before being changed to an oral supplement, which raises questions as to adherence to protocol.
• Counterintuitively, mild hypomagnesemia required a longer correction period compared to severe cases, potentially pointing to differences in treatment approaches and patient responses
• The deviation from the recommended duration of treatment with intravenous magnesium sulphate may indicate that Trust's guidelines require revision to show the realities of clinical practice.
• The audit highlighted the need for further education of clinicians regarding the management of hypomagnesemia, particularly on the importance of daily monitoring and following through with the treatment protocols.

## Introduction

Hypomagnesaemia is a common electrolyte disturbance among patients admitted to Scarborough General Hospital. Hypomagnesaemia, while typically defined as having serum magnesium concentration below 0.7 mmol/L (1.7 mg/dL), with or without accompanying total body depletion, does not lead to clinically significant signs and symptoms until serum levels fall below 0.5 mmol/L (1.2 mg/dL) giving way to life-threatening features such as dysrhythmia, muscle weakness and haemodynamic instability.^1^ Nonetheless, as magnesium is involved in an array of structural and physiological functions, adverse effects associated with hypomagnesaemia may occur in almost every organ system, whether they are clinically acute and overt, or chronic and subtle. Hypomagnesaemia is rarely in isolation; it tends to co-exist with other electrolyte abnormalities, including but not limited to hypokalaemia, hypophosphataemia or hypocalcaemia. It contributes significantly to morbidity, thus if not addressed promptly, it delays patient recovery, subsequently prolonging hospital stay.^2^

York and Scarborough Teaching Hospitals NHS Foundation Trust drafted guidelines to help in diagnosis and management of hypomagnesaemia. In this report, we throw more light on the practices at Scarborough General Hospital vis-a-vis the standards set out in the above guidelines. Are they being followed? Do we need more sensitisation or adjustment to the guidelines? We hope that the results of this audit can give an answer to this question in addition to making recommendations to continue improving management of patients with hypomagnesaemia.

## Aims and standards

To determine whether trust protocols are followed in diagnosis and management of hypomagnesaemia. This audit aimed to evaluate the management of hypomagnesaemia at Scarborough General Hospital, focusing on adherence to trust guidelines and identifying areas for improvement in clinical practice.

## Methodology

A clinical audit proforma was filled in by the patients, and retrospective data were collected from the electronic patient record system. Definitions were derived from the trust protocol, which defines mild hypomagnesaemia as serum magnesium levels ranging from 0.5 to 0.69 mmol/L, and moderate to severe hypomagnesaemia as levels below 0.5 mmol/L (see Appendix A). Data analysis was performed using Microsoft Excel. The audit was registered within the trust clinical audit department (AUD018).

## Results

We conducted an audit of 98 patients with hypomagnesaemia in the time period between May and December 2023. The mean age of the participants was 74.6 ± 15.1 years, with slight female predominance, see Table 1. Approximately two-thirds of the participants had mild hypomagnesaemia, with 10 patients having ECG changes. Gastrointestinal cause accounted for most cases of hypomagnesaemia, and seven patients had eGFR < 30. The results of this audit are summarised in Table 1.

**Table 1:** Baseline patient characteristics.

Characteristic	Value (%)
Mean age ± SD	74.6 ± 15.1
Gender	
- Males	46 (46.9)
- Females	52 (53.1)
Severity of hypomagnesaemia	
- Mild	67 (67.3)
- Moderate/severe	31 (31.6)
Patients with symptomatic hypomagnesaemia	34 (36.2)
Patients with ECG done	65 (66.3)
Patients with ECG changes	10 (13.7)
Causes of hypomagnesaemia	
- Medications	55 (24.5)
- Gastrointestinal	47 (48.0)
- Renal	24 (24.5)
Patients with CKD (eGFR < 30)	7 (7.1)

Most patients with hypomagnesaemia are asymptomatic. This was clearly demonstrated from data collected, which revealed that only 36% (34) of patients were symptomatic with features of hypomagnesaemia. This could be explained by the fact that 67% (67) patients only have mild hypomagnesaemia. However, 24% (24) had severe symptomatic hypomagnesaemia, and this needs prompt correction to avert life-threatening features.

To continue with, the trust guidelines advise for ECG checks for patients found to have hypomagnesaemia. 66% (65) patients had ECGs done, but only 10% (10) were found to have ECG changes. ECG changes commonly start to appear in very severe cases and thus recognition of such features should prompt urgent action. Cardiac monitoring is thus very important when severe hypomagnesaemia is detected.

In addition, the most common causes of hypomagnesaemia were medications and gastrointestinal causes. We found use of proton pump inhibitors, especially lansoprazole and omeprazole, to be a particularly common culprit among the medications. Judicious use of proton pump inhibitors could in theory help reduce hypomagnesaemia, especially in older patients. We also noted that alcohol, reduced oral intake and dehydration were quite common gastrointestinal causes of hypomagnesaemia.

Further still, in Table 2 below, it is observed that only a small number of patients presented with severe and/or life-threatening hypomagnesaemia. All were subjected to cardiac monitoring as recommended by the trust guidelines, but only half had documented treatment with intravenous magnesium sulphate. No patient ever needed to be given intravenous magnesium sulphate for the entire 3 days as recommended. One dose was always adequate, and the majority were switched to oral therapy the following day after initiation of parenteral treatment.

**Table 2:** Breakdown of patients with severe/symptomatic hypomagnesaemia (n=24).

Characteristic	Value (%)
Patients with life-threatening complications	8 (33.3)
Patients with cardiac monitoring	9 (37.5)
Patients treated with IV magnesium sulphate	4 (17.4)
Mean duration of therapy ± SD (hours)	4.1 ± 2.3
Patients with oral magnesium supplementation	9 (37.5)

About 53 patients (80.6%) received oral magnesium supplementation. Among them, 18 patients (26.9%) were administered the supplement once daily, while 35 patients (52.2%) received it twice daily. In addition, we observed that, on average, it took about 3.5 days to correct severe hypomagnesaemia, while 4.5 days were needed for mild forms. The difference in duration of resolution of hypomagnesaemia isn't much; however, we note that parenteral administration of magnesium sulphate in severe cases helps to cut down significantly on time taken to correct hypomagnesaemia. One potential explanation could be put down to the pharmacokinetics of magnesium supplementation, including the rate of absorption and bioavailability, differs between oral and intravenous routes. Intravenous magnesium leads to faster and more predictable increases in serum magnesium levels, while oral magnesium, depending on the patient's gastrointestinal absorption capability, may take longer to correct the deficiency, especially in those with gastrointestinal issues. However, oral magnesium replacement should be considered first, as a sudden rise in serum magnesium concentration (as seen following intravenous replacement) partially removes the stimulus for magnesium retention, and up to 50% of the infused magnesium is excreted in the urine.^3^ Magnesium sulphate has a high osmolarity and may cause tissue damage if it extravasates into the surrounding tissue.^3^

**Fig. 1:**
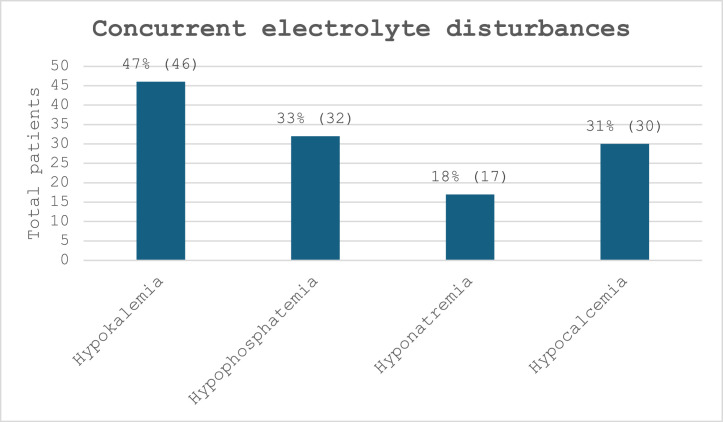
Bar chart showing the frequency of concurrent electrolyte disturbances among patients.

As depicted in the trust guidelines, hypomagnesaemia rarely occurs on its own. We noted that hypokalaemia was found to be the most common co-existing electrolyte imbalance, but hypophosphataemia and hypocalcaemia too were quite frequent (Fig. 1). This therefore re-emphasises the importance of checking for co-existing electrolyte disorders once hypomagnesaemia is found.

In this audit, we noted that in 91% (89) of the cases, calcium and phosphate levels were checked. This was particularly done in patients in the emergency department, but less among patients on the medical wards found to have hypomagnesaemia. This is an area for improvement. In addition, only 66% (65) of patients with hypomagnesaemia had magnesium levels monitored daily. This is still significantly low, and thus more sensitisation is needed to improve this practice.

## Conclusion

The trust guidelines are quite comprehensive in terms of guiding clinicians on how to safely correct hypomagenesaemia and identify the possible causes. Areas of acceptable performance were: (1) ECG checking was done for the majority of patients; (2) other electrolyte imbalances were identified and corrected; (3) patients with severe hypomagnesaemia were subjected to cardiac monitoring; and (4) magnesium levels were promptly corrected in those with severe/symptomatic hypomagnesaemia. Meanwhile, areas that needed improvement were: (1) daily monitoring of magnesium levels; (2) documentation of identified causes; (3) monitoring of magnesium levels, especially after parenteral treatment; and (4) standardisation of the duration of administration of parenteral magnesium sulphate. The trust guidelines recommend 3 full days of treatment with parenteral magnesium sulphate, but we noted that this was never required to correct severe and/or symptomatic hypomagnesaemia. One dose of parenteral magnesium sulphate followed by oral switch, monitoring and correction of other electrolyte imbalances was usually sufficient to correct severe hypomagnesaemia. Taking this into account, a revisit to this part of the guidelines should be considered. In addition, the guidelines recommend 6 h parenteral intravenous magnesium sulphate. However, we noted varying treatment duration from as short as 30 min to 6-hour treatment durations. More sensitisation among clinicians about the trust guidelines on diagnosis and management of hypomagnesaemia is still needed. This can be achieved through organising teaching sessions on hypomagnesaemia targeting all doctors in the trust, and distribution of the step-by-step flow chart on hypomagnesaemia management to key areas like the emergency department, acute medical unit, care of the elderly and endocrinology wards.

## Data availability

The data that support the findings of this study are available from the corresponding author, DG, upon reasonable request.

## Ethical approval and consent to participate

This audit was registered with the Clinical Audit Department under reference number AUD018. In accordance with local guidelines, ethical approval was not required for this project as it was conducted as a clinical audit. The purpose of the audit was to evaluate the quality of care within the institution, and no interventions outside standard clinical practice were introduced.

As the audit involved the analysis of anonymised patient data, individual consent to participate was not required. The principles of confidentiality and data protection were adhered to throughout the audit process.

## Funding

This research did not receive any specific grant from funding agencies in the public, commercial, or not-for-profit sectors

## CRediT authorship contribution statement

**Dhanushan Gnanendran:** Writing – review & editing, Writing – original draft, Data curation. **Moses Grace Kintu:** Methodology, Conceptualization. **Aparna Ravikumar:** Visualization, Investigation. **Tadeusz Pawlak:** Supervision.

## Declaration of competing interest

The authors declare that they have no known competing financial interests or personal relationships that could have appeared to influence the work reported in this paper.

## Figures and Tables

**Figure:**
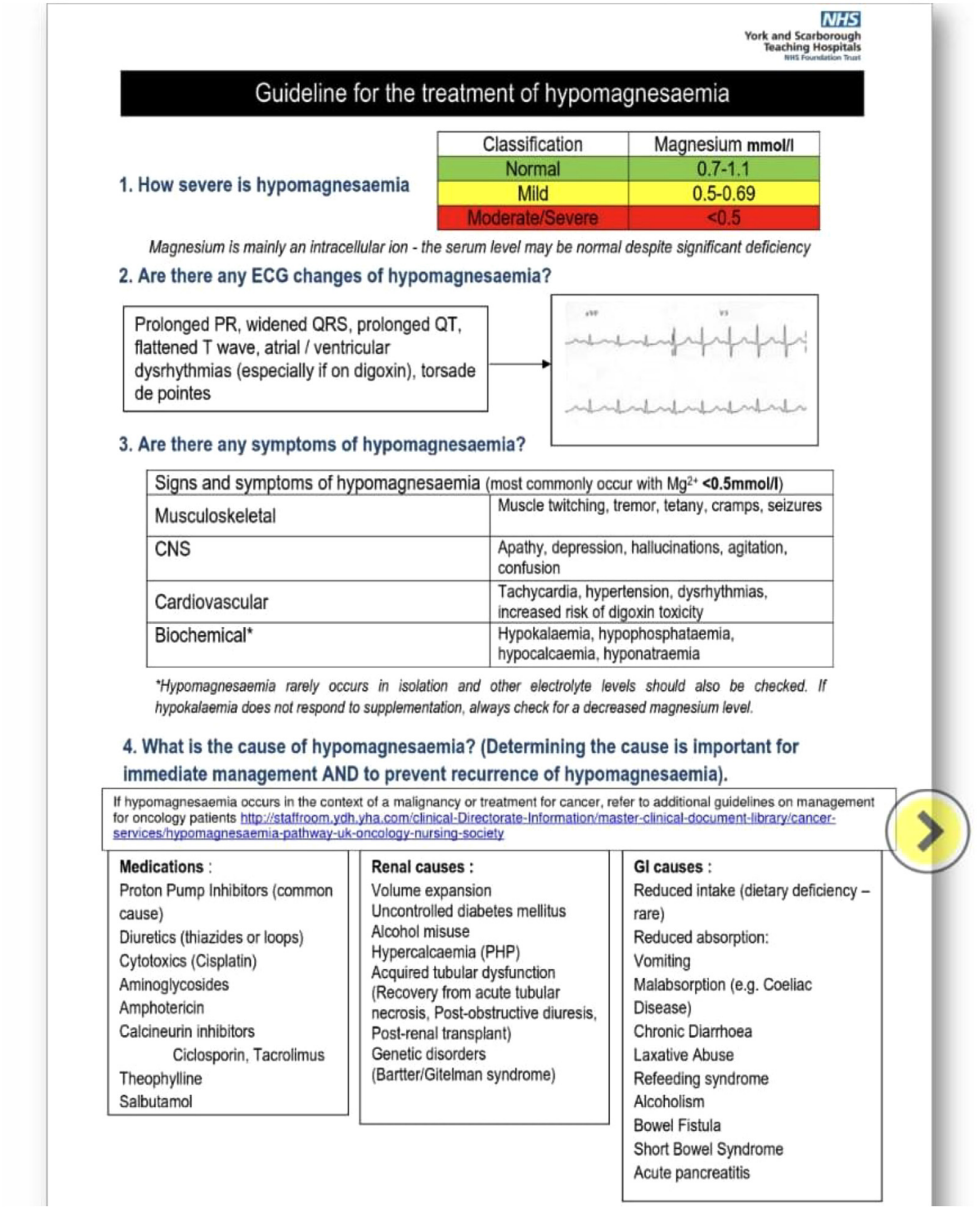


**Figure:**